# Goitrogenic Anions, Thyroid-Stimulating Hormone, and Thyroid Hormone in Infants

**DOI:** 10.1289/ehp.0901736

**Published:** 2010-05-03

**Authors:** Yang Cao, Benjamin C. Blount, Liza Valentin-Blasini, Judy C. Bernbaum, Terry M. Phillips, Walter J. Rogan

**Affiliations:** 1 Epidemiology Branch, National Institute of Environmental Health Sciences, National Institutes of Health, Department of Health and Human Services, Research Triangle Park, North Carolina, USA; 2 Department of Health Statistics, Faculty of Health Services, Second Military Medical University, Shanghai, China; 3 Division of Laboratory Sciences, National Center for Environmental Health, Centers for Disease Control and Prevention, Atlanta, Georgia, USA; 4 Children’s Hospital of Philadelphia, Philadelphia, Pennsylvania, USA; 5 Ultramicro Immunodiagnostics Laboratory, National Institute of Biomedical Imaging and Bioengineering, Bethesda, Maryland, USA

**Keywords:** infant, iodide, nitrate, perchlorate, thiocyanate, thyrotropin, thyroxine

## Abstract

**Background:**

Environmental exposure of infants to perchlorate, thiocyanate, nitrate, might interfere with thyroid function. U.S. women with higher background perchlorate exposure have higher thyroid-stimulating hormone (TSH) and lower thyroxine (T_4_). There are no studies with individual measures of thyroid function and these goitrogens available in infants.

**Objective:**

We examined the association of urinary perchlorate, nitrate, iodide, and thiocyanate with urinary T_4_ and TSH in infants and whether that association differed by sex or iodide status.

**Methods:**

We used data and samples from the Study of Estrogen Activity and Development, which assessed hormone levels of full-term infants over the first 12 months of life. The study included 92 full-term infants between birth and 1 year of age seen up to four times. Perchlorate, thiocyanate, nitrate, and iodide were measured in 206 urine samples; TSH and T_4_ and were measured in urines and in 50 blood samples.

**Results:**

In separate mixed models, adjusting for creatinine, age, sex, and body mass index, infants with higher urinary perchlorate, nitrate or thiocyanate had higher urinary TSH. With all three modeled, children with higher nitrate and thiocyanate had higher TSH, but higher perchlorate was associated with TSH only in children with low iodide. Unexpectedly, exposure to the three chemicals was generally associated with higher T_4_.

**Conclusions:**

The association of perchlorate exposure with increased urinary TSH in infants with low urinary iodide is consistent with previous findings. Higher thiocyanate and nitrate exposure were also associated with higher TSH in infants.

Perchlorate (ClO_4_^−^) is an inorganic anion used industrially as an oxidizer for rocket fuels and propellants and in explosives. Perchlorate can also form naturally in the atmosphere and accumulate in arid regions. It has become a widespread environmental contaminant (Wilson 2008). Perchlorate was detected in 4% of U.S. public drinking water samples, with detectable levels ranging from 4 μg/L to 440 μg/L. It is estimated that > 1 million people have perchlorate in their public drinking water supplies at levels of at least 4 ppb, based on sampling data collected by the U.S. Environmental Protection Agency as of May 2004 (National Research Council 2005). Perchlorate has also been detected in dairy milk as well as a variety of other foods. In a nationwide survey, the U.S. Food and Drug Administration detected perchlorate in at least one sample of 74% of the foods analyzed (Murray et al. 2008). Widespread human exposure to perchlorate was recently reported from the National Health and Nutrition Examination Survey (NHANES) (2001–2002); all 2,820 spot urine specimens analyzed contained perchlorate, and the median urine perchlorate in the U.S. population was 3.6 μg/g creatinine (Blount et al. 2006). Perchlorate competitively inhibits iodide uptake, and high-dose exposure will decrease thyroid function (Kirk 2006; Wolff 1998). Perchlorate was used to treat Graves’ disease in the 1950s and 1960s (Godley and Stanbury 1954) and is still used to treat amiodarone-induced thyrotoxicosis (Martino et al. 2001).

Adequate thyroid hormone production is critical in pregnant women and neonates, because thyroid hormone is required for mental development in children (American Academy of Pediatrics et al. 2006). Based on a controlled dosing study of seven healthy adults, a perchlorate dose of 6.4 μg/kg/day was estimated to produce no effect on 24-hr radioactive iodine uptake (Greer et al. 2002). Exposure models predict that infants are unlikely to be exposed to doses this high (Baier-Anderson et al. 2006); however, infants may be more sensitive than adults to thyroid inhibition (Lawrence et al. 2000), thus raising the possibility of an effect on thyroid function in infants from background exposure to perchlorate. In addition, infants may have inadequate iodine intake or may be exposed to thiocyanate from tobacco smoke (Gasparoni et al. 1998) or to nitrate from drinking water (Manassaram et al. 2006). Thiocyanate and nitrate can also inhibit iodine uptake in the thyroid (Tonacchera et al. 2004), and a low intake of iodine might increase the likelihood of a perchlorate effect. To date, only ecological studies of perchlorate exposure and thyroid function in infants are available. Amitai et al. (2007) found no difference in neonatal thyroxine (T_4_) levels despite maternal consumption of drinking water that contained high perchlorate concentrations. Buffler et al. (2006) did not observe an association between estimated average perchlorate concentrations > 5 μg/L in drinking water supplies and high thyroid-stimulating hormone (TSH) concentrations. In contrast, Brechner et al. reported associations between perchlorate exposure and elevated TSH levels in newborns (Brechner et al. 2000; Kelsh et al. 2003), commented on by Lamm (2003). All of these analyses were retrospective, without direct measurements of perchlorate or iodide in the infants. The conflicting results of these studies and their lack of individual assessment of perchlorate and other relevant exposures emphasize the need for further evaluation of perchlorate exposure and thyroid function in infants.

The objective of our study was to see whether urinary perchlorate was associated with T_4_ and TSH in infants. More specifically, we wanted to know whether any such association was stronger in those with lower iodide status and in females and thus consistent with NHANES 2001–2002 data showing an association between higher urinary perchlorate, higher serum TSH, and lower serum T_4_ in females ≥ 12 years of age who had lower iodide status (Blount et al. 2006). We also wanted to examine the role of thiocyanate and nitrate, because both are common exposures in infancy.

## Methods

### Study participants

We used samples and data already collected in the Study of Estrogen Activity and Development (SEAD). The detailed design of the SEAD was published elsewhere (Cao et al. 2009). SEAD was a cross-sectional study that enrolled children according to age, sex, and diet so that samples and data were collected from groups of 12 children (six girls and six boys, four of whom were fed breast milk, four cow milk formula, and four soy formula) representing 31 different ages (< 48 hr of age, at weekly intervals from 1 week to 23 weeks of age, and at monthly intervals from 6 months to 12 months of age). Individual children could participate at up to four different ages, yielding 166 unique infants. The study was cleared by the institutional review boards at the Hospital of the University of Pennsylvania and the Children’s Hospital of Philadelphia, where recruitment and clinical visits took place, and at the National Institute of Environmental Health Sciences. The children were eligible if they had been born at term (37–41 weeks), with birth weight 2,500–4,500 g, and free of any major illness or birth defects. The feeding group definitions were stricter for the younger children and relaxed for the older children. Urine samples were also collected at all visits, resulting in 381 urine samples (nine more than called for by the design because of over-recruitment) (Cao et al. 2009) representing 165 children. Blood samples were collected at 88 visits. At each visit, study staff measured the child’s height, weight, and head circumference. The SEAD study focused on sex hormones, but T_4_ and TSH were also measured in all samples. We had used a layered consent for any use of the samples beyond what was planned in the original study and thus had to recontact families to ask permission to do further chemical analysis on 177 samples from 84 children.

### Laboratory methods

As part of SEAD, blood and urinary TSH and T_4_ were measured by the Ultramicro Immunodiagnostics Laboratory at the National Institute of Biomedical Imaging and Bioengineering, National Institutes of Health (Bethesda, MD), by recycling immunoaffinity chromatography using an array of capillary immunoaffinity columns packed with antibody-coated glass beads (Phillips 2001). Each column contained a single, specific antibody and isolated its specific analyte, allowing the sample to pass to the next column. In this way, all analytes could be isolated from the same sample during the same run. The specificity of each antibody was immunochemically checked by two-dimensional Western blotting against all of the other analytes prior to use. Bound analytes were labeled with laser dye and detected by laser-induced fluorescence using a scanning detector and a fiberoptic spectrometer. The concentrations of each analyte were calculated by comparison with standard curves constructed by running known amounts of each analyte through the array under the same conditions. Additionally, the analytes from each column were collected and subjected to characterization by mass spectrometry. The limits of detection (LODs) in whole blood were 1.1 μIU/mL and 0.1 ng/dL for TSH and free T_4_, respectively, and 1.8 μIU/mL and 0.8 ng/dL for the same analytes in urine. Intra-assay coefficients of variance (CV) for the two analytes were 2.9% and 3.5% for TSH and free T_4_ in whole blood and 4.2% and 5.1% for the same analytes in urine. The interassay CVs for TSH and free T_4_ were 4.6%, 5.5% in whole blood and 5.2%, 6.1% in urine. Comparison of the recycling immunoaffinity system with conventional enzyme immunoassays demonstrated *r*^2^ values of 0.981 and 0.977 for the measurement of TSH and free T_4_ in spiked human whole blood and 0.973 and 0.971 for TSH and free T_4_ in spiked human urine.

For the current study, urine samples were shipped on dry ice to the Division of Laboratory Sciences, National Center for Environmental Health, Centers for Disease Control and Prevention (CDC; Atlanta, GA) and analyzed for perchlorate, thiocyanate, nitrate, and iodide using ion chromatography tandem mass spectrometry (Blount et al. 2007; Valentin-Blasini et al. 2005). Reported results for all specimen met the division’s quality control and quality assurance performance criteria for accuracy and precision. The LODs for perchlorate, nitrate, thiocyanate, and iodide were 0.05, 500, 10.0, and 0.33 μg/L, respectively. To adjust for fluctuations in urine concentration, the urinary analyte concentrations were also expressed as mass per mass creatinine.

Urine samples were collected either by urine bag or by compressing a wet cloth diaper. We suspected that cloth diapers might have perchlorate extractable by urine. We thus wet lot–matched diaper material (*n* = 9) with 10 mL deionized water, cut out the wet part, compressed it in a 50-mL syringe, and analyzed the expressed water.

To make an independent assessment of lab quality control at the CDC lab, we randomly selected 11 (5%) of the samples, split them, and submitted them twice for analysis; they were not labeled as replicates. The results are an indication of reproducibility, which we evaluated by using the intraclass correlation coefficient (ICC).

### Statistical methods and data analysis

Descriptive results were expressed as means or geometric means and 95% confidence intervals (CIs) for continuous variables, and by percentages for categorical variables. For any samples reported as below the LOD, we substituted a value of 
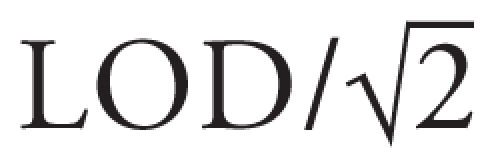
. We transformed analyte concentrations when necessary to achieve symmetric, approximately Gaussian distributions of regression residuals. Residuals for all concentrations were more symmetric after natural log transformation. The descriptive results were adjusted for age by a mixed linear model. To examine the correlations between analytes or between matrices, we used Spearman’s correlation coefficient.

The SEAD study was partly cross-sectional and partly longitudinal. Because some infants had up to four visits during the study and may have more than one measure of exposure and thyroid function, the calculation of standard errors and *p*-values took into account any correlations among multiple visits by the same infant. We used a mixed linear model that accounted for correlations among several measurements of the same subject. Candidates for covariates for the regression analysis were age, race/ethnicity, length, weight, head circumference, feeding method, and sex. Analyses were conducted by Stata 10.1 software (StataCorp, College Station, TX, USA) and SAS 9.13 software (SAS Institute Inc., Cary, NC, USA).

The geometric mean urinary concentrations of perchlorate, iodide, and thiocyanate differed by feeding method, and TSH and T_4_ by feeding method and sex. Except for nitrate, there was no difference by race in the concentrations of the analytes (Table 1). Thus, we did not include race as a covariate in the further mixed linear model analyses.

The covariates that we chose for the multivariate mixed linear model analyses are known to be or likely to be associated with T_4_ or TSH. These covariates were age, sex, urinary thiocyanate, urinary nitrate, and urinary iodide. Although feeding method—breast milk or formula—might affect thyroid function, we had no prior evidence that it did. Breast-feeding, however, increases exposure to perchlorate (Valentin-Blasini L, Blount BC, Otero-Santos S, Bernbaum JC, Cao Y, Rogan WJ, unpublished data) and introducing such an exposure variable into the statistical model would tend to bias, usually by decreasing, the coefficient for perchlorate (Greenland et al. 1999). Thus we did not include feeding method in the models. In adults, using creatinine-specific concentrations when modeling effects of environmental contaminants can introduce bias if creatinine per se is associated with the outcome variable (Barr et al. 2005), in our case TSH and T_4_. We found very small, nonsignificant correlations between creatinine and TSH and T_4_ (Spearman correlation coefficients between urine creatinine and TSH and T_4_ are −0.04 and −0.001, respectively), and thus used the creatinine-specific concentrations in the models.

TSH and T_4_ are usually measured in blood or serum, but we had relatively few such samples; of the 206 urine samples, only 50 had blood samples from the same children at the same visit. When we limited our analysis to only those samples, there was no significant association between TSH or T_4_ and perchlorate, thiocyanate, and nitrate exposures (data not shown). However, because of the small sample size, we had very low power to test our hypotheses. We did find correlations, however, between those blood concentrations we did have in the larger SEAD and urine concentrations of TSH and T_4_. The Spearman’s correlation coefficients for TSH and T_4_ between the two matrices were 0.49 and 0.56 (*p* < 0.05 for both). We thus chose to base our analyses on the urine concentrations.

## Results

We obtained consent for this study from the families of 92 of the original 165 infants and analyzed the 206 samples we had from them (Table 2). Of the 92 infants, 41 contributed samples once, 14 twice, 11 three times, and 26 four times. We considered a *p*-value of < 0.05 to indicate statistical significance. There were no statistically significant differences in child’s sex, age, or body mass index (BMI) between those infants whose mothers gave consent to analyze samples for this study and those whose mothers did not (data not shown). There were differences for race and feeding method. More than 95% of white mothers consented to sample analysis, but only 39% non-Hispanic black mothers did so (χ^2^ = 43.4; *p* < 0.01). Of mothers who breast-fed, 79% gave consent; of mothers who did not, 45% gave consent (χ^2^ = 17.5; *p* < 0.01). All of these infants had TSH and T_4_ measured in urine, and 42 of them also had TSH and T_4_ measured in blood.

Initially, all urine samples had detectable levels of perchlorate. However, perchlorate was present in the water extracted from the control cloth diapers (1.24 ± 0.23 μg/L), so we subtracted 1.24 μg/L from the concentration found in all samples (*n* = 164) that had been collected using the diapers. This reduced 16% of the samples to below the LOD for perchlorate. No other analyte was detected in the diapers. Iodide was detected in all 206 urine samples, but the levels of thiocyanate and nitrate were below the LOD in 13 (6%) and 29 (14%) samples, respectively. The intraclass correlation coefficients of duplicate measurements for 11 pairs of samples for iodide, perchlorate, thiocyanate, and nitrate were all above 0.95, which demonstrates high reproducibility of the laboratory analyses. The demographic characteristics and results of the laboratory analyses are shown in Table 3.

The mixed linear model analysis that included only one chemical exposure at a time (but also included age and BMI as continuous variables and sex as covariates) showed that perchlorate, thiocyanate, and nitrate all had significantly positive associations with TSH and T_4_ (Table 4, “Unique chemical” columns). The mixed linear model that included covariates as above and perchlorate, thiocyanate, and nitrate simultaneously showed that thiocyanate and nitrate were significantly associated with TSH and that thiocyanate was associated with T_4_ (Table 4, “Multiple chemicals” columns). In general, associations between TSH and thiocyanate were larger than those between TSH and perchlorate or nitrate. For example, in the model of TSH and the individual chemicals (Table 4, “Unique chemical” TSH), doubling urinary perchlorate or nitrate would increase the TSH estimate by about 10%, but doubling urinary thiocyanate would increase it by about 20%.

We searched for statistically significant effect estimates for perchlorate after stratifying by sex and iodide status. We measured urinary iodide, the predominant form of iodine present in urine (Valentin-Blasini et al. 2007), and used 100 μg/L as the cut point of iodide groups, because it is a simple, close approximation of the 100-μg/L iodine cut point used by the World Health Organization (WHO) to indicate iodine deficiency [WHO, United Nations International Children’s Fund (UNICEF), Integrated Center for Child Development (ICDD) 2001]. This yielded 75 measurements from 48 children < 100 μg/L and 131 measurements from 73 children ≥ 100 μg/L. For mean iodide level, there are 28 children < 100 μg/L and 64 children ≥ 100 μg/L. For sex, we found no significant association in either boys or girls between perchlorate and TSH or T_4_ (Table 5). We found that among children with lower iodide, those with higher perchlorate had statistically significantly higher TSH but not statistically significantly higher T_4_ (Table 5). There were too few children to examine differences in slopes in boys and girls in strata of iodide excretion.

## Discussion

We measured TSH, T_4_, perchlorate, thiocyanate, nitrate, and iodide concentrations in urine of individual infants from birth to 12 months of age. Other factors that may affect thyroid function were also measured. In models including clinical variables and perchlorate or thiocyanate or nitrate concentrations, we found a significant positive association between each of the goitrogenic anions and urinary TSH and T_4_. When we included all three goitrogenic anions in one model, we found that thiocyanate and nitrate remained significant, whereas the perchlorate term did not, and its effect estimate decreased to close to the null. When we tested the prior hypothesis that any perchlorate effect would be seen in children with lower iodide, we found a significant increase in TSH in children with lower iodide and high perchlorate, even if we included the nitrate and thiocyanate terms, and no evidence of an effect in those with higher iodide. We did not confirm our prior hypothesis that girls would be more susceptible than boys.

The strengths of this study include having individual measurements of perchlorate, TSH, and T_4_, clinical information, and measurements of the two other common environmental chemicals that operate through a similar mechanism as perchlorate–nitrate and thiocyanate. This study is the first to assess thyroid function in infants with this assay and spot urines. The method we used to measure thyroid hormone is new, but, as noted above, it demonstrated very high *r*^2^ values with the accepted enzyme immunoassays. There is experimental evidence that the kidney is the major route of clearance for TSH (Constant and Weintraub 1986). In adults, TSH can be reliably detected in urine, is higher in patients with hypothyroidism, and is increased in the nephrotic syndrome and other illnesses in which urinary protein is high (Yoshida et al. 1988). Also in adults, T_4_ is readily detected in urine and is higher in patients with hyperthyroidism; the authors of the paper stated that they thought that 24-hr excretion of T_4_ was a good indicator of thyroid function (Orden et al. 1987). We found no reports of the use of urine for TSH and T_4_ analysis after the 1980s. We speculate that this was due to advances in analytical technique for small samples of serum and blood, making them the matrix of choice, because we found no reports showing substantial error of the measurements in urine. There is thus some general albeit older support for the use of TSH and T_4_ in urine. If the physiologically important measure is blood TSH or T_4_, and urine provides an imprecise measure of it that is not affected by the exposure variable, then the effect is to shrink observed associations and reduce power. At these exposures, it seems very unlikely that any of these anions would have an important but previously undescribed effect on kidney function or metabolism. The lack of perfect correlation between urinary and blood concentrations per se cannot produce associations that are not there. The most plausible way for an artifactual association to arise would be through an association of the contaminant chemicals with urinary creatinine, which has been described in adults (Barr et al. 2005). However, there is no such association in these relatively isosthenuric infants, and we can think of no other straightforward way in which using urinary TSH would produce an association with perchlorate.

Unexpectedly, among children with low iodide in urine, the infants with higher perchlorate and TSH do not have lower T_4_ levels. However, the relationship between T_4_ and TSH in young infants is less stable than in adults. In the first 3 months after birth, high T_4_ can be accompanied by normal TSH (Bursell and Warner 2007). Although this observation does not explain the finding, it supports the idea that a clear relation between higher T_4_ and lower TSH may not be seen as readily in young infants as it is in adults. We attempted to test this hypothesis simply, by replacing the BMI term with a marker term for age > 12 weeks and < 12 weeks. The marker term was not significant, and its presence made almost no change in the other model coefficients (data not shown), which we interpret to mean that the data set is too small to examine this hypothesis in detail. There is an experimental study in deer mice exposed perinatally to perchlorate that showed increased serum T_4_, which the authors thought might be explained by an increase in the glandular surface area due to functional iodine deficiency and thus a greater sensitivity to TSH (Thuett et al. 2002). Finally, although hypothyroid patients have lower urinary T_4_, their renal clearance of T_4_ is higher, and we could speculate that this may account for some of the unexpected occurrence of higher TSH in the presence of higher T_4_ (Orden et al. 1987).

These data show association between several goitrogenic anions found in the environment and a measure of thyroid function in children recruited from general pediatric clinics. Their perchlorate exposure likely comes from a combination of sources, including drinking water, infant formula, and breast milk (Pearce et al. 2007; Schier et al. 2009) (Valentin-Blasini L, Blount BC, Otero-Santos S, Bernbaum JC, Cao Y, Rogan WJ, unpublished data). The potential effects of perchlorate and other inhibitors of iodine uptake should be reduced by adequate intake of iodine; however, iodine intake has decreased in the United States, raising concerns about adequate intake in pregnant and lactating women (Hollowell and Haddow 2007). The average urinary thiocyanate level in these infants was almost 50 times higher than the average perchlorate level, and thiocyanate is more strongly related to TSH than perchlorate. Thiocyanate has been measured in bovine milk and human milk and exposure in children could result from consumption of cyanogenic foods or exposure to tobacco smoke (Gasparoni et al. 1998). Our findings provide one more reason not to smoke around infants. For children this age, nitrate comes from drinking water, although vegetables such as spinach and squash can contribute to their nitrate exposure (Greer et al. 2005). The infants in this study lived in an urban environment, so they likely drank city water with low levels of nitrate. Further work is needed to determine whether such exposures have any effect on thyroid function. Finally, the unexpected finding that the chemical exposures associated with higher TSH were also associated with higher T_4_ needs further study

## Conclusion

The weak but significant association we find between higher urinary perchlorate and higher urinary TSH in infants with lower urinary iodide is consistent with previous findings in U.S. women and with the expected effect of perchlorate. Further study is needed to confirm the association, to explore the unexpected association with higher T_4_, and to investigate whether the same association is found with TSH in serum.

## Figures and Tables

**Table 1:** Geometric mean concentrations of urinary analytes per gram creatinine (95% CI) by feeding method, sex, and race.^a^

	Perchlorate (μg/g cr)	Iodide (μg/g cr)	Nitrate (mg/g cr)	Thiocyanate (μg/g cr)	TSH (mIU/g cr)	T_4_ (μg/g cr)
Feeding method						
Breast milk	28.0 (19.2–40.6)*	1,400 (1,100–1,700)*	95 (69–131)	1,000 (760–1,320)*	41 (32–52)*	0.17 (0.12–0.22)*
Cow formula	9.5 (5.7–15.6)*	1,100 (900–1,500)	108 (70–167)	620 (430–900)*	26 (18–36)	0.09 (0.06–0.14)
Soy formula (reference)	2.5 (1.6–3.8)	800 (600–1,000)	123 (84–180)	200 (140–270)	23 (17–31)	0.08 (0.06–0.12)

Sex						
Boy	10.1 (7.2–14.2)	1,100 (900–1,300)	125 (93–167)	550 (430–710)	24 (19–30)*	0.08 (0.06–0.11)*
Girl (reference)	7.5 (5.2–10.7)	1,000 (800–1,200)	94 (69–128)	440 (340–580)	35 (27–44)	0.14 (0.11–0.19)

Race						
Other	9.0 (6.1–13.1)	1,100 (900–1,400)	140 (100–195)*	500 (370–650)	32 (25–42)	0.11 (0.08–0.14)
Non-Hispanic black (reference)	8.4 (6.0–11.9)	1,000 (800–1,200)	84 (62–112)	500 (380–640)	26 (20–33)	0.11 (0.08–0.15)

cr, creatinine.

a Adjusted for age, BMI, feeding method, sex, and race; values < LOD were replaced by 
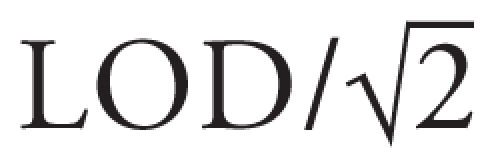
; mixed linear models account for multiple measures in the same child.

* *p* < 0.05.

**Table 2:** Nominal age range at collection of 206 urine samples analyzed for goitrogenic anions and iodide.^a^

Nominal age	Range (days)	Boy	Girl	Total
Newborn	< 2	5	4	9
1 week	2–9	2	4	6
2 weeks	10–16	3	2	5
3 weeks	17–23	4	3	7
4 weeks	24–30	1	2	3
5 weeks	31–37	3	3	6
6 weeks	38–44	3	2	5
7 weeks	45–51	4	3	7
8 weeks	52–58	4	2	6
9 weeks	59–65	4	3	7
10 weeks	66–72	5	4	9
11 weeks	73–79	3	2	5
12 weeks	80–86	2	6	8
13 weeks	87–93	3	4	7
14 weeks	94–100	4	3	7
15 weeks	101–107	5	5	10
16 weeks	108–114	3	1	4
17 weeks	115–121	3	4	7
18 weeks	122–128	5	3	8
19 weeks	129–135	5	3	8
20 weeks	136–142	3	3	6
21 weeks	143–150	2	4	6
22 weeks	151–156	3	3	6
23 weeks	157–163	4	3	7
6 months	164–181	4	3	7
7 months	182–209	3	3	6
8 months	210–238	4	4	8
9 months	239–265	4	3	7
10 months	266–293	4	4	8
11 months	294–321	3	3	6
12 months	322–378	4	1	5
Total		109	97	206

a Ninety-two unique infants participating at up to four nominal ages.

**Table 3:** Demographic characteristics and analyte concentrations.

Variable	Children (*n*)	Sample (*n*)	Arithmetic mean (95% CI)	Geometric mean (95% CI)	Percent
Age (days)	92	206	122 (104–140)		
BMI (kg/m^2^)	92	206	16.2 (15.8–16.6)		
Sex					
Boy	47				51
Girl	45				49
Race/ethnicity					
Non-Hispanic black	44				48
Other	48				52
Feeding method					
Breast milk	42				46
Cow milk formula	22				24
Soy formula	28				30
Blood analyte^a^					
TSH (μIU/mL)	42	50		5.2 (4.4–6.1)	
Free T_4_ (ng/dL)	42	50		3.3 (2.7–4.0)	
Urinary analyte^a^					
TSH (μIU/mL)	92	206	4.2 (3.7–4.6)		
Free T_4_ (ng/dL)	92	206		1.4 (1.2–1.6)	
Iodide (μg/L)	92	206		130.3 (112.8–150.5)	
Perchlorate (μg/L)	92	206		1.21 (0.86–1.69)	
Thiocyanate (μg/L)	92	206		67.3 (53.3–84.9)	
Nitrate (μg/L)	92	206		12,100 (9,420–15,530)	
Creatinine (mg/dL)	91	205		11.8 (10.5–13.3)	
Urinary analyte (mass/g cr)^a^					
TSH (mIU/g cr)	91	205		29.6 (24.6–35.6)	
Free T_4_ (μg/g cr)	91	205		0.11 (0.09–0.14)	
Iodide (μg/g cr)	91	205		1,080 (930–1,250)	
Perchlorate (μg/g cr)	91	205		9.8 (7.0–13.8)	
Thiocyanate (μg/g cr)	91	205		540 (430–690)	
Nitrate (mg/g cr)	91	205		106 (85.2–132)	

cr, creatinine.

a Adjusted for age; values < LOD were replaced by LOD/16% for perchlorate; 0% for iodide; 6% for thiocyanate; 14% for nitrate. Mixed linear models account for multiple measures in the same child.

**Table 4:** Coefficients (95% CI) of mixed-model analyses of urine TSH and T4 on iodide, perchlorate, thiocyanate and nitrate.

	Log (TSH, mIU/g cr)		Log (T_4_, μg/g cr)	
Independent variable	Unique chemical^a^	Multiple chemicals^b^	Unique chemical^a^	Multiple chemicals^b^
Log (iodide, μg/g cr)	0.44 (0.28 to 0.59)*		0.39 (0.22 to 0.55)*	
Log (perchlorate, μg/g cr)	0.12 (0.05 to 0.19)*	0.01 (−0.07 to 0.09)	0.11 (0.03 to 0.19)*	0.01 (−0.08 to 0.09)
Log (thiocyanate, μg/g cr)	0.28 (0.17 to 0.39)*	0.26 (0.15 to 0.37)*	0.34 (0.23 to 0.45)*	0.33 (0.21 to 0.44)*
Log (nitrate, μg/g cr)	0.15 (0.07 to 0.23)*	0.13 (0.05 to 0.21)*	0.11 (0.02 to 0.19)*	0.09 (0.01 to 0.17)*

cr, creatinine. All models are adjusted for age, sex, and BMI.

a Results for individual chemicals modeled separately.

b Results for models with all chemicals modeled simultaneously.

* *p* < 0.05.

**Table 5:** Coefficients (95% CI) of mixed-model analyses of urine TSH and T4 on interaction between perchlorate and sex or iodide.

	Log (TSH, mIU/g cr)		Log (T_4_, μg/g cr)	
Independent variable	Stratum-specific coefficients boy or girl	Stratum-specific coefficients < or ≥100 μg/L iodide	Stratum-specific coefficients boy or girl	Stratum-specific coefficients < or ≥100 μg/L iodide
Log (thiocyanate, μg/g cr)	0.27 (0.16 to 0.38)*	0.28 (0.17 to 0.39)*	0.34 (0.22 to 0.46)*	0.34 (0.23 to 0.46)*
Log (nitrate, μg/g cr)	0.13 (0.05 to 0.21)*	0.13 (0.05 to 0.21)*	0.08 (−0.01 to 0.17)	0.08 (0.01 to 0.16)*
Log (perchlorate, μg/g cr)				
Boy	−0.06 (−0.16 to 0.04)		−0.09 (−0.20 to 0.03)	
Girl	0.04 (−0.05 to 0.13)		0.04 (−0.05 to 0.14)	
Log (perchlorate, μg/g cr)				
Iodide < 100 μg/L		0.10 (0.01 to 0.19)*		0.09 (−0.01 to 0.19)**
Iodide ≥ 100 μg/L		−0.04 (−0.12 to 0.04)		−0.05 (−0.14 to 0.04)

cr, creatinine. Mixed models account for multiple measures in the same child. All models are adjusted for age and BMI; models with sex-specific estimates are not adjusted for sex.

* *p* < 0.05.

** *p* < 0.1.
