# Errata

**Published:** 2010-11

Cao et al. [ Environ Health Perspect 118:1332–1337 (2010)] have reported errors in their paper. On page 1333 in the second paragraph of “Statistical methods and data analysis,” the following sentence was omitted:

Because length, weight, and head circumference were highly correlated with age (the pairwise Pearson correlation coefficients were 0.91, 0.84, and 0.83, respectively), they were not included in the model, but we combined length and weight as body mass index (BMI) and introduced it into the model.

In the fourth paragraph of that section, one of the covariates (BMI) was omitted. The corrected sentence is as follows:

These covariates were age, sex, BMI, urinary thiocyanate, urinary nitrate, and urinary iodide.

In addition, in the footnote of Table 3, “LOD/16%” was incorrect. The corrected sentence is as follows:

Adjusted for age; values < LOD were replaced by 
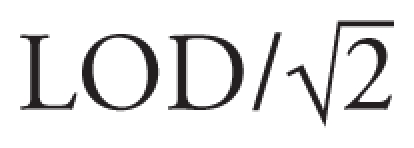
; 16% for perchlorate; 0% for iodide; 6% for thiocyanate; 14% for nitrate. Mixed linear models account for multiple measures in the same child.

